# Genetic Determinants of Cardiovascular Disease: The Endothelial Nitric Oxide Synthase 3 (eNOS3), Krüppel-Like Factor-14 (KLF-14), Methylenetetrahydrofolate Reductase (MTHFR), MiRNAs27a and Their Association with the Predisposition and Susceptibility to Coronary Artery Disease

**DOI:** 10.3390/life12111905

**Published:** 2022-11-16

**Authors:** Rashid Mir, Imadeldin Elfaki, Jamsheed Javid, Jameel Barnawi, Malik A. Altayar, Salem Owaid Albalawi, Mohammed M. Jalal, Faris J. Tayeb, Aadil Yousif, Mohammad Fahad Ullah, Faisel M. AbuDuhier

**Affiliations:** 1 Prince Fahd Bin Sultan Research Chair, Department of Medical Lab Technology, Faculty of Applied Medical Sciences, University of Tabuk, Tabuk 71491, Saudi Arabia; 2 Department of Biochemistry, Faculty of Science, University of Tabuk, Tabuk 71491, Saudi Arabia; 3 Department of Cardiology, King Fahd Specialist Hospital, Tabuk 71491, Saudi Arabia

**Keywords:** coronary artery disease (CAD), endothelial nitric oxide synthase 3 (eNOS), Krüppel-like factor-14 (KLF-14), methylenetetrahydrofolate reductase (MTHFR), MicroRNA, tetra-primer amplification refractory mutation system (T-ARMS), Hardy–Weinberg disequilibrium (HWD)

## Abstract

Coronary artery disease (CAD) is an important cause of death worldwide. CAD is caused by genetic and other factors including hypertension, hyperlipidemia, obesity, stress, unhealthy diet, physical inactively, smoking and Type 2 diabetes (T2D). The genome wide association studies (GWASs) have revealed the association of many loci with risk to diseases such as cancers, T2D and CAD. Nitric oxide (NO) is a potent vasodilator and is required for normal vascular health. It is produced in the endothelial cells in a reaction catalyzed by the endothelial NO synthase (eNOS). Methylenetetrahydrofolate reductase (MTHFR) is a very important enzyme involved in metabolism of folate and homocysteine, and its reduced function leads to cardiovascular disease. The Krüppel-like factor-14 (KLF-14) is an important transcriptional regulator that has been implicated in metabolic syndrome. MicroRNA (MiRNAs) are short non-coding RNAs that regulate the gene expression of proteins involved in important physiological processes including cell cycle and metabolism. In the present study, we have investigated the potential impact of germline pathogenic variants of endothelial eNOS, KLF-14, MTHFR, MiRNA-27a and their association with risk to CAD in the Saudi population. Methods: Amplification Refractory Mutation System (ARMS) PCR was used to detect MTHFR, KLF-14, miRNA-27a and eNOS3 genotyping in CAD patients and healthy controls. About 125 CAD cases and 125 controls were enrolled in this study and statistical associations were calculated including *p*-value, risk ratio (RR), and odds ratio (OD). Results: There were statistically significant differences (*p* < 0.05) in genotype distributions of MTHFR 677 C>T, KLF-14 rs972283 G>A, miRNAs27a rs895819 A>G and eNOS3 rs1799983 G>T between CAD patients and controls. In addition, our results indicated that the MTHFR-TT genotype was associated with increased CAD susceptibility with an OR 2.75 (95%) and *p* < 0.049, and the KLF14-AA genotype was also associated with increased CAD susceptibility with an OR of 2.24 (95%) and *p* < 0.024. Moreover, the miRNAs27a-GG genotype protects from CAD risk with an OR = 0.31 (0.016), *p* = 0.016. Our results also indicated that eNOS3 -GT genotype is associated with CAD susceptibility with an OR = 2.65, and *p* < 0.0003. Conclusion: The MTHFR 677C>T, KLF14 rs972283 G>A, miRNAs27a A>G, and eNOS3 rs1799983 G>T genotypes were associated with CAD susceptibility (*p* < 0.05). These findings require verification in future large-scale population based studies before these loci are used for the prediction and identification of individuals at risk to CAD. Weight control, physical activity, and smoking cessation are very influential recommendations given by clinicians to the at risk individuals to reduce or delay the development of CAD.

## 1. Introduction

Cardiovascular disease (CVD) is a very important cause of morbidity and death worldwide including the kingdom of Saudi Arabia (KSA) [1]. In 2019, the WHO estimated that CVD was the cause of 17.9 million deaths which represented more than 30% of all deaths in that year. It has been reported that in the Gulf Cooperation Council which including the KSA, about 45% of deaths are caused by CVD [2]. Coronary artery disease (CAD) is one of the important types of CVD [3]. CVD is caused by lifestyle and genetic risk factors [4]. Lifestyle risk factors include unhealthy diet, physical inactivity, obesity, smoking and type 2 diabetes (T2D) [4]. The genome wide association studies have indicated the association of certain loci with CAD, diabetes, and other diseases in different populations [5,6,7,8,9,10,11,12,13,14,15].

Nitric oxide (NO) is an important signaling molecule with a short half-life in body fluids [16]. NO is a crucial mediator of endothelial function [17,18]. It is synthesized in the vascular endothelial cells in a reaction catalyzed by endothelial NO synthase (eNOS) [17,18]. Physiological production of NO by eNOS has been linked to a healthy cardiovascular system, and reduced bioavailability of NO has been associated with CVD [17,18]. NO maintains the homeostasis of cardiovascular cells and has anti-hypertensive properties. Moreover, NO protects vasculature from white blood cell immigration and adhesion as well as platelet aggregation [17]. Single nucleotide variations (SNVs) of eNOS gene were associated with diseases such as Multiple sclerosis and uterine cervical cancer in Iranian and Chinese populations, respectively [19,20]. Recently, the eNOS rs1799983 SNP were associated elevated susceptibility to hypertension [21].

The role of transcription factors is crucial. Krüppel-like factor 14 (KLF-14) is one of the members of the Krüppel-like family (KLF) and is a transcription factor from the third group of KLFs that inhibits the transcription of genes by binding to the deoxynucleic acid with Sin3A [22]. The latter is a transcriptional co-suppressor [22]. KLF-14 is an important metabolic transcriptional regulator that has been implicated in metabolic syndrome [22,23]. KLF-14 protects against the inflammatory process in endothelial cells via the inhibition of signaling pathway of NF-κB [24]. KLF-14rs972283 SNV was reported to be associated with T2D [25]. Moreover, SNVs in KLF-14 gene were associated with lipid profiles, status of blood pressure, resistance to insulin and metabolic syndrome [26].

MTHFR is a very important enzyme involved in the metabolism of folate, homocysteine, nucleotide synthesis, and methylation of membranes, DNA, protein and lipids [27,28]. Increased blood levels of homocysteine have been demonstrated to be associated with increased risk to CVD [29]. The C677T SNV is found at exon 4 which replaces valine to alanine at codon 222 [30], and decreases the catalytic activity of MTHFR [30]. Furthermore, the MTHFR 677 SNV was reported to be associated with psoriasis, CAD, diabetes, and neurological disease [30,31].

MicroRNAs are short noncoding RNA molecules that modulate the expression of genes and are involved in many pathophysiological processes [32]. MiR-27a regulates Peroxisome proliferator-activated receptor Gamma (PPARγ) [33]. PPARγ has been implicated in CVD [34]. The miRNAs27a rs895819 SNV was reported to be associated with susceptibility to myocardial infarction (MI) and breast cancer in the Chinese population [35,36].

The genome wide association studies (GWAS) have helped in uncovering the linkage between certain loci and diseases including diabetes, CVD, cancers, and others [5,8,9,10,13,14,37]. In the current study, we investigated the association of pathogenic gene variants of eNOS rs1799983, KLF14rs972283, MTHFR 677, and miR-27a rs895819 with the risk to CAD in the Saudi Arabian population.

## 2. Materials and Methods

This study addresses clinically confirmed cases of CAD in the population from the Tabuk region, Saudi Arabia. This project is approved by the ethics committee, University of Tabuk (No: UT-91-23-2020). Informed consent was obtained before collection of samples from all participants.

### 2.1. Patient Selection Criteria

#### 2.1.1. Criteria for Inclusion of Subjects in this Study

We selected cases conducting elective angiography for the diagnosis of stable angina at the King Fahad Specialist Hospital, KSA. Other tests were also conducted such as electrocardiogram (ECG or EKG), ambulatory electrocardiography, Holter monitoring, X-ray, echocardiogram (echo) for chest, cardiac computed tomography (CCT), test for exercise stress and myocardial perfusion imaging (MPI) or Multigated acquisition scan (MUGA).

The subjects were divided according to the result of the coronary angiographic to either significant coronary artery disease or ischemic heart disease (stenosis ≥ 50%) or no ischemic heart disease (no stenosis or stenosis < 50%). Cases with cancers, diabetes, or other chronic disorders have been excluded from this project.

#### 2.1.2. Healthy Controls

Healthy controls were subjects attending King Fahd Specialist Hospital for regular checkups. The healthy controls were apparently healthy with no history of cardiovascular disease, or any other chronic diseases. Blood biochemistry tests were also done for the healthy controls. All subjects filled the informed consent form.

### 2.2. Genomic DNA Extraction

The Genomic DNA from each subject was purified with the DNeasy Blood K (Qiagen, Hilden, Germany) according to the manufacturer’s protocol. The DNA amount was quantified with the NanoDrop, and DNA integrity was assessed with agarose gel electrophoresis. Then DNA was kept at 25 °C till genotyping was performed.

#### 2.2.1. Genotyping of *MTHFR* 677, KLF-14, miRNAs27a and eNOS3 SNPs

The genotyping of *MTHFR* 677, KLF-14, miRNAs 27a and eNOS3 was determined by ARMS-PCR using tetra-primers (Table 1).

#### 2.2.2. Preparation of PCR Mix

The PCR reaction was conducted in 12 µL that contained sample DNA (50 ng), F_O_—0.10 µL, R_O_—0.10 µL, FI—0.10 µL, RI—0.10 µL (25 pmol of desired primer), and 6 µL Green Master Mix for PCR (2X) (Cat# M712C, Promega, Madison, WI, USA). Then water free from nuclease was added to bring the total volume to 12 µL.

#### 2.2.3. PCR Conditions

The conditions were: initial denaturation at 95 °C (08 min), then 32 cycles of denaturation at 94 °C (34 s); annealing temperatures for 44 s MTHFR 677 C>T (58 °C), KLF 14 C>T (60 °C), miRNAs27a A>G (63 °C), eNOS3-G>T (60 °C) for 45 s, extension at 72 °C for 35 s, and a final extension 72 °C for 10 min.

#### 2.2.4. Gel Electrophoresis and PCR Product Visualization

Products of the PCR were run in agarose gel electrophoresis (1.5%) stained with SYBR safe dye. The gel was visualized on a UV transilluminator from Bio-Rad, Hercules, CA, USA.

#### 2.2.5. MTHFR 677 C>T rs1801133 Genotyping

The external primers F0 and R0 amplify the external region of the MTHFR gene, producing a band of 224 bp that serves as a control for DNA soundness. Primers F0 and RI amplify the T allele, producing a band of 177 bp, and primers FI and Ro generate a band of 101 bp from the C allele

#### 2.2.6. KLF-14 rs972283 G>A Genotyping

The external primers F1 and R1 amplify the external region of the KLF-14, producing a band of 437 bp that serves as a control for DNA soundness. Primers FO and RI amplify the A allele, producing a band of 221 bp, and primers FI and RO producing a band of 274 bp from the G allele.

#### 2.2.7. MiRNAs27ars895819 A>G Genotyping

The primers F0 and R0 amplify the external region of the miR-27, producing a band of 353 bp that is used as a control for DNA soundness. Primers F0 and RI amplify the A allele, giving a band of 226 bp, and primers FI and R0 produce a band of 184 bp from the G allele.

#### 2.2.8. eNOS3-rs1799983 (Glu298Asp) G>T Genotyping

Primers F1 and R1 flank the exon of the eNOS3, producing a band of 701 bp that serves as a control for DNA integrity. Primers F0 and RI amplify the T allele, producing a band of 271 bp, and primers FI and RO produce a band of 475 bp from the T allele.

### 2.3. Statistical Analyses

The differences of genotype distributions of the SNPS MTHFR 677 C>T, KLF-14 rs972283 C>T, miR-27a rs895819 A>G and eNOS3-rs1799983 G>T in CAD subjects and controls were calculated with Chi-square test, and checked with Hardy–Weinberg equilibrium (HWE). The associations between the genotypes and alleles of the SNVs and the risk to CAD were estimated by the odds ratios (ORs), risk ratios (RRs) and risk differences (RDs) with 95% confidence intervals (CIs). A *p*-value < 0.05 was regarded as statistically significant. All statistical analyses were conducted with SPSS 16.0.

## 3. Results

### 3.1. Demographic Features of CAD Patients

Table 2 summarizes the demographic trends observed in 125 consecutive CAD patients. From a group having 125 CAD profiles, the clinical data was collected for all 125 cases available; however, patients were stratified based on age into above 50 years (*n* = 30, 24%) or ≤50 years (*n* = 95, 76%) as summarized in Table 2. Out of 125 CAD cases, 84 were males and 41 females

Lipid biomarkers: Results indicated that 52% had cholesterol higher than 200 mg/dL, 40.8% had LDL levels higher than 100 mg/dL, 56% had HDL levels higher than 40 mg/dL, whereas 36% had TGL higher than 150 mg/dL as depicted in Table 2. About 40.8% CAD patients had higher level of serum creatinine >1.35 mg/dL and 57.6% CAD patients had higher level of serum C-reactive protein > 10 mg/L. Out of 125 CAD cases, 41.6% were having hypertension, 44% had T2D (+), 32% had obesity and 57.6% had MI. Out of 125 cases, 56% of subjects were smokers.

### 3.2. Hardy–Weinberg Equilibrium for Genotype Distributions and Allele Frequencies

There was no deviation observed for the genotype distributions and allele frequencies of the SNPs for the four genes: MTHFR 677 C>T, KLF 14 rs972283 C>T, miR-27a rs895819 A>G and eNOS3-rs1799983 G>T in the control group. Therefore, the genotyping results were reviewed from the 10% of the samples randomly chosen from the normal control group, which reflected the accuracy rate of more than 99%.

### 3.3. Statistical Analysis of MTHFR 677 C>T, KLF 14G>A, miRNAs27a-A>G and eNOS3G>T Genotypes with CAD Patient Susceptibility

At the time of analysis, KLF-14-rs972283 G>A, eNOS3-rs1799983 G>T and miRNAs27ars895819 A>G SNP were studied in 125 CAD cases and 125 controls, and MTHFR 677 C>T gene polymorphism was analyzed in 116 CAD cases and 125 healthy controls.

### 3.4. Comparative Statistical Analysis of MTHFR 677 C>T Genotypes Assessed for CAD Patients and Controls

The MTHFR 677 C>T genotype frequency in CAD patients and controls was CC (57.75%), CT (37.93%) and TT (12%) and controls CC (63.2%), CT (24.8%) and TT (4.48%) respectively (Table 3). The results for MTHFR 677 C>T gene variation was statistically significant (*p* < 0.045) between CAD patients and controls. It was also noted that the T allele had a higher frequency among CAD patients than in healthy individuals (0.31 vs. 0.17) (Table 3).

### 3.5. The Association between MTHFR 677 C>T Genotypes and Risk to Patients with CAD Cases Estimated through Multivariate Analysis Based on OR and RR (CI = 95%)

As summarized in Table 4, our results demonstrated that in the codominant model, the MTHFR-TT genotype was associated strongly with increased CAD patient susceptibility with an OR of 2.75, (95%) CI = (1.0018 to 7.5558), RR = 1.80 (0.9084 to 3.5814), and *p* < 0.049; whereas no association with CAD susceptibility was reported for the MTHFR-CT genotype with an OR of 1.67, (95%) CI = (0.9529 to 2.9393), and *p* < 0.73. We also observed a significant association between the MTHFR-CC and MTHFR-(CT+TT) genotypes in the dominant inheritance model that may be linked to a greater CAD susceptibility with an OR = 1.84, (95% CI) (1.0930 to 3.1257), and *p* < 0.021. Moreover, no association was found between the MTHFR-(CT+TT) and MTHFR-TT genotypes in the recessive inheritance model. The allelic comparison found the MTHFR -T allele to be significantly associated with CAD susceptibility with an OR of 1.82, (95% CI) (1.1816 to 2.8041), RR 1.39 (1.0777 to 1.813), and *p* < 0.006 (Table 4).

### 3.6. The Association of MTHFR 677 C>T Genotypes with CAD Patient Characteristics

There was no significant association between MTHFR 677 C>T genotypes assessed for the age and gender of the CAD patients (*p*>0.05), as reported in Table 5. However, the association between MTHFR 677 C>T genotypes and serum cholesterol (mg/dL) of CAD patients was significant (*p* = 0.003). In addition, a significant association was found between MTHFR 677 C>T genotypes and LDL-C (mg/dL) of CAD patients (*p* < 0.0018). Moreover, HDL-C (mg/dL) of CAD patients did not show significant association with MTHFR 677 C>T genotypes (*p* = 0.560). Similarly, the statistical analysis of the correlation between MTHFR 677 C>T genotypes and creatinine (mg/dL) in CAD patients did not show any significant association (*p* =0.313). MTHFR 677 C>T genotypes and CRP (mg/L) in CAD patients revealed a significant association (*p* < 0.033). A significant correlation of MTHFR 677 C>T genotypes was however observed with hypertension (*p* = 0.002), T2D (*p* = 0.0067), obesity (*p* < 0.022) and MI (*p* = 0.0001), which was also the case with smoking (*p* = 0.005) in CAD patients (Table 5).

### 3.7. Association of KLF-14 rs972283 G>A Genotypes between Cases and Controls

The frequency of KLF-14 rs972283 G>A genotypes in CAD patients and controls was GG (46.4%), GA (22.4%) and AA (31.2%) and controls GG (40%), GA (48%) and AA (12%), respectively, as reported in Table 6. The KLF-14 rs972283 G>A polymorphic gene variation found between cases and healthy individuals was statistically significant (*p* < 0.045). It was reported that the allele A had a higher frequency among CAD patients than in healthy individuals (0.43 vs. 0.36) as shown in Table 6.

### 3.8. Multivariate Analysis Predicting the Association of KLF-14 rs972283 G>A Genotypes with the Risk to Patients with CAD Cases

This analysis is based on a logistic regression-like odd ratio (OD), and a risk ratio (RR) with 95% CI was used to estimate the association between the KLF14 rs972283 G>A genotypes and risk of CAD patients—the data are summarized in (Table 7). As reported in the codominant model, the KLF14-AA genotype was demonstrated to be associated with increased CAD susceptibility with an OR of 2.24, (95%) CI = (1.1070 to 4.5383), RR = 1.66 (1.0358 to 2.6817), and *p* < 0.024; whereas the KLF14-GA genotype was shown to be a potential protective marker for CAD risk with an OR 0.40, (95%) CI = (0.2237 to 0.7234), and *p* < 0.002. Our study found no association between the KLF14-GG and KLF14 (GA+AA) genotypes in the dominant inheritance model with an OR = 0.77 and *p* < 0.30. Interestingly, a strong association also existed between the KLF14-(GG+GA) and KLF14-AA genotypes in the recessive inheritance model with an OR of 3.32, (95%) CI = (1.7207 to 6.4275), RR = 2.02 (1.2914 to 3.1608), and *p* = 0.0004. The KLF-14-A allele was not associated with CAD susceptibility with an OR of 1.30 and *p* < 0.14 (Table 7).

### 3.9. The Association of KLF-14 rs972283 G>A Genotypes with CAD Patients Demographic and Clinical Variables

As displayed in Table 8, there was no association between KLF-14 rs972283 G>A genotypes and the age and gender of the CAD patients (*p* > 0.05). However, the analysis of the association between KLF-14 G>A genotypes and serum cholesterol (mg/dL) of CAD patients showed a significant association (*p =* 0.0032). Similar results were found for KLF-14 G>A genotypes and its association with serum LDL-C (mg/dL) of CAD cases (*p* < 0.002). Moreover, our results showed that there was no significant association between KLF-14 G>A genotypes and serum HDL-C (mg/dL) of CAD patients with a (*p =* 0.26). On the other hand, a significant association was found between KLF-14 G>A genotypes and serum triglyceride TG (mg/dL) of CAD patients, with *p =* 0.008. The statistical analysis of the correlation between KLF-14 G>A genotypes and CRP < 10 mg/L in CAD patients demonstrated a significant association (*p* = 0.0001). A significant correlation was also found between KLF-14 G>A genotypes and hypertension (*p* = 0.002), T2D (*p* = 0.0002) and MI (*p* = 0.009). As mentioned, no significant correlation existed between KLF-14 G>A genotypes and obesity (*p* = 0.10) in CAD patients (Table 8).

### 3.10. Association of miRNAs27a rs895819 A>G Genotypes in a Correlation Study between CAD Cases and Healthy Individual Controls

The frequency of *miRNAs27a* rs895819 A>G genotypes in CAD cases and healthy individuals was GG (52%), GA (42.4%) and AA (5.6%), and controls GG (44.8%), GA (40%) and AA (15.2%), respectively (Table 9). The miR-27a rs895819 A>G gene polymorphic variations analyzed for CAD cases and controls was reported to be statistically significant (*p* < 0.032). As shown in Table 9, the frequency of the allele A was higher among CAD cases than in than in healthy individuals (0.73 vs. 0.65).

### 3.11. Multivariate Analysis for Predicting the Association between miRNAs27a rs895819 A>G Genotypes and Risk to Patients with CAD Cases

This analysis is based on a logistic regression-like OR or risk ratio RR with 95% CI and was used to estimate the association between the miR-27a rs895819 A>G genotypes and risk of CAD patients. As reported in Table 10, we found that in the codominant model, the miRNAs27a-GA genotype was not associated with CAD susceptibility with an OR of 0.91, (95%) CI = (0.5669 to 1.5038), RR = 0.95 (0.7258 to 1.2501), and *p* < 0.738; whereas miR-27a-GG genotype has a significance of protective biomarker for CAD risk with an OR 0.31, (95%) CI = (0.1243 to 0.8104), and *p* < 0.016. There is no association observed between miRNAs27a-AA and miRNA27a-(GA+GG) genotypes in the dominant inheritance model with an OR = 0.74 and *p* < 0.25. The miRNA27a-GG vs (AA+GA) genotypes in the recessive inheritance model also acts a protective biomarker for CAD susceptibility with an OR of 0.33, (95%) CI = (0.1338 to 0.8184), RR = 0.62(0.4801 to 0.8076) *p* = 0.016. In addition, in allelic comparison, miRNA27a-G allele acts as a protective biomarker against the CAD susceptibility with an OR of 0.67 and *p* < 0.04 (Table 10).

### 3.12. The Association of miRNAs27a rs895819 A>G Genotypes with CAD Patients Demographic and Clinical Variables

The study found no significant association between miRNA27a rs895819 A>G genotypes with either the age or the gender of the CAD patients (*p* > 0.05), as shown in Table 11. By contrast, we did find the association between miRNA27a A>G genotypes and serum cholesterol (mg/dL) of CAD patients (*p* = 0.046). Similarly, a significant association between miRNA27a A>G genotypes and serum LDL-C (mg/dL) of CAD patients was also reported (*p* < 0.006). However, no association between miRNA27a A>G genotypes and serum HDL-C (mg/dL) was reported for CAD patients (*p* = 0.66). A significant association between miR-27a A>G genotypes and serum TAGS (mg/dL) of CAD patients have been reported (*p* = 0.0019). The statistical analysis of the correlation between miRNA27a A>G genotypes and C-reactive protein < 10 mg/L in CAD patients revealed a significant association (*p* = 0.121). A significant correlation was found between miRNA27a A>G genotypes and hypertension (*p* = 0.0001), T2D (*p* = 0.0001) and MI (*p* = 0.005). Further the study did not find any correlation between miRNA27a A>G genotypes and obesity (*p* = 0.49) in CAD patients (Table 11).

### 3.13. Association of eNOS3-rs1799983 G>T Genotypes in the Comparative Study between CAD Cases and Controls

The eNOS3-rs1799983 G>T genotypes frequency was GG (30.4%), GT (65.6%) and TT (4%) in CAD patients, and for controls were GG (50.4%), GT (41.6%) and TT (8%) as shown in Table 12. The miR-27a rs895819 A>G polymorphic gene variation exhibited between CAD cases and healthy individuals in the control group was statistically significant (*p* < 0.0007). In addition, as shown in Table 12, the frequency of the allele A was higher among CAD patients than in healthy individuals (0.37 vs. 0.29).

### 3.14. Multivariate Analysis to Estimate the Association between eNOS3-rs1799983 G>T Genotypes and Risk to Patients with CAD Cases

This analysis was based on a logistic regressionlike OR, and RR with 95% CI was calculated to estimate the association between the eNOS3-rs1799983 G>T genotypes and risk of CAD patients as displayed in Table 13. The study found that in the codominant model, the eNOS3-GT genotype was associated strongly with CAD susceptibility with an OR of 2.65, (95%) CI = (1.5619 to 4.5162), RR = 1.61(1.2468 to 2.0969), and *p* < 0.0003; whereas the eNOS3-TT genotype did not show any association with CAD risk with an OR 0.93, (95%) CI = (0.292 to 2.99), and *p* < 0.74. Additionally, a strong association was still observed between the eNOS3-GG and eNOS3-(GT+TT) genotypes in the dominant inheritance model with an OR = 2.40 and *p* < 0.0009. There was no association observed between the eNOS3-(GG+GT) and eNOS3-TT genotypes in the recessive inheritance model with an OR of 0.53, (95%) CI = (0.174 to 1.650), RR = 0.76 (0.506 to 1.153). The allelic comparison found allele eNOS3–T was associated strongly with the susceptibility to CAD with an OR of 1.49 and *p* < 0.036 (Table 13).

### 3.15. Association of eNOS3-rs1799983 (Glu298Asp) G>T Genotypes with Demographic and Clinical Variables for CAD Patients

The study showed no significant association between eNOS3-rs1799983 G>T genotypes with either the age or the gender of the CAD patients (*p* > 0.05), as reported in Table 14. The statistical analysis exhibited a significant association between eNOS3 G>T genotypes and serum cholesterol (mg/dL) in CAD patients (*p =* 0.031). Similarly, a significant association between eNOS3 G>T genotypes and serum TAGs (mg/dL) in CAD patients was also reported (*p =* 0.014). There was no significant association reported between eNOS3 G>T genotypes and serum HDL-C (mg/dL)/serum LDL-C (mg/dL) in CAD patients (*p =* 0.07 and 0.07, respectively). The statistical analysis of the correlation between eNOS3 G>T genotypes and CRP < 10 mg/L in CAD patients displayed a significant association (*p* =0.0008).

A significant correlation was found between eNOS3 G>T genotypes and hypertension (*p* = 0.0001), T2D (*p* = 0.029) and MI (*p =* 0.002). There was also a significant correlation reported between eNOS3 G>T genotypes and obesity (*p* = 0.004) in CAD patients (Table 14).

## 4. Discussion

CVD including CAD is an important cause of death worldwide. Our results showed that the MTHFR 677 C>T SNV was associated with increased CAD risk (Table 2 and Table 3). This result is consistent with previous investigations [31,38,39]. The MTHFR is crucial for metabolism of folate, replication of DNA, and methylation of DNA and proteins [40]. It is required for synthesis of proteins and nucleic acids and for cellular homeostasis [39]. The 5,10-methylenetetrahydrofolate is converted to 5-methyltetrahydrofolate by the MTHFR [41]. In the synthesis of protein, the 5-methyltetrahydrofolate donates the CH_3_ to convert the homocysteine to methionine [42]. It has been reported that the C677T C>T SNV reduces the activity of the MTHFR enzyme [39,43]. In addition, it has been reported that increased serum homocysteine is a risk factor for CVD in young cases [38,44]. The increased serum homocysteine is accompanied by oxidative stress, stimulates the aggregation of platelet, and induces the dysfunction of endothelial cells, increasing hypercoagulability, stress of endoplasmic reticulum and the proliferation of blood vessel smooth muscular cells [38,44]. These lead to atherosclerosis and CAD [44].

Our results showed that there was a significant difference in MTHFR 677 C>T SNV in cases with hyperlipidemia and cases with normal lipid profile (Table 4). Hyperlipidemia is a classical risk factor for CAD [45,46]. Our results indicated that the MTHFR 677 C>T genotype distribution is significantly different between cases with increased and cases with normal CRP (Table 5). The CRP is an inflammatory marker, and elevated levels of CRP were associated with CAD [47]. The role of inflammation in induction of atherosclerosis and CAD is well-established [48]. Previous studies conducted in mice have reported that the reduced MTHFR can alter inflammatory responses and lipid metabolism in hepatocytes [49,50]. We also found a significant difference in the MTHFR 677 C>T SNV genotype distribution in cases with and cases without T2D (Table 5). Indeed, such an association of MTHFR 677 C>T SNV with T2D and diabetic nephropathy have also been observed in Asian populations [51,52]. MTHFR is very important in metabolism of folate and involved in diabetes and its complications [51,53]. As shown in the results, there was a significant difference in the MTHFR 677 C>T SNV genotype distribution in cases with and cases without hypertension (Table 5). Similarly, in the Irish population, the association of MTHFR 677 TT genotype with increased blood pressure independently of homocysteine has been reported [54], and additionally, the treatment with the riboflavin that is a co-factor of MTHFR can reduce blood pressure in hypertensive cases [54]. The hypertension leads to CAD [55].

Our result indicated that the KLF-14 rs972283 G>A SNV was associated with CAD (Table 3 and Table 4). The potential role the KLF-14 rs972283 G>A SNV in the induction of CVD (through dyslipidemia) has also been shown in the Chinese population [56]. A recent study has reported the association of KLF-14 SNVs with metabolic syndrome such as CVD and Type 2 diabetes (T2D) [22]. It has been reported that the KLF14 mediates signaling of lipid and that SNVs in KLF genes influence the KLF roles in maturation of adipocytes, in the hepatocytes (lipid metabolism), in regulation of blood vessels and in angiogenesis [22]. Moreover, it has been demonstrated that the KLF-14 expression might be involved in the formation of atherosclerotic lesion [57]. A significant difference in KLF-14 rs972283 G>A genotype distribution has been observed in patients with normal lipid profile compared to patients with hyperlipidemia (Table 8). Furthermore, significant differences were also seen in patients without T2D and MI compared with patients with T2D and MI (Table 7). Studies have supported the role of KLF-14 genetic variants in induction of metabolic syndrome including obesity, insulin resistance, T2D and CVD [22]. This is since the KLF-14 (and other KLF members) regulates the metabolism of glucose and lipid and is involved in adipogenesis, in differentiation and in the function of adipocytes [22]. Results also showed that there was a significant difference in KLF-14 rs972283 G>A SNV between cases with normal and those with elevated CRP (Table 8). This result is expected hence increased CRP is a biomarker of CVD. The CRP enhances the inflammation and the damage of the blood vessels [58]. It has been reported that normal expression of KLF-14 suppresses the transcription of the signaling pathway of NF-kappa-B and thereby inhibits the inflammation in vascular endothelium mediated by the macrophages [22]. Moreover, KLF-14 prevents endothelial cells from the inflammatory stresses [22]. The role of blood vessel inflammation in the development of CVD is well-established [48].

Our results showed that the GG genotype and the G allele of the miRNAs27a rs895819 A>G were associated with reduced risk to CAD (Table 5 and Table 6). A study indicating the association of A allele of the miRNAs27a rs895819A>G with increased risk to MI has been reported in the Chinese Han population wherein the role of high-density lipoprotein-cholesterol (HDL-C levels) has been noticed [35]. The miRNAs27a rs895819 A>G SNV is a loop-stem structure SNV influencing the function of the mature miRNAs27a [59]. It has been reported that the G allele of the miR-27a rs895819 was associated with decreased expression of the mature miRNAs27a [59]. Our results indicated that the miRNAs27a rs895819 A>G genotype distribution was significantly different between cases with normal lipid levels and those with hyperlipidemia (Table 11). It is known that the metabolism of lipids is also regulated by miRNAs27a through the targeting of the nuclear receptor, retinoid X receptor alpha, which has a role in adipogenesis [60,61]. MiRNAs27a also reported targeting the ATP-binding cassette sub-family A member1 (ABCA1) which is involved in the metabolism of cholesterol [61,62]. Results also showed that there was significant difference in miRNAs27a rs895819 A>G genotype distribution in cases with and those without T2D (Table 11). A previous study in an in vivo model has reported that miRNAs27a regulates the PPAR-γ-PI3K/AKT-GLUT4 signaling axis and has an active role in insulin signaling and T2D development [63]. Our results are also consistent with a study conducted in the Iranian population which reported that the C allele of the miRNAs27a rs895819 confers protection against T2D [64].

As shown, the eNOS3-rs1799983 G>T SNV was associated with CAD (Table 12 and Table 13), which has also been indicated by some previous investigations [21]. The eNOS is an important effector of the cGMP pathway and produces nitric oxide (NO) by converting L-arginine to L-citrulline [65]. The Genes (eNOS) encoding the endothelial NOS was reported as an important gene for cardiovascular physiology since it is crucial for the enzyme activity of the NOS and NO production [21,66]. The NO is a blood vessels relaxing factor produced by the endothelial cells [16]. It is a vasodilator regulating the blood pressure, blood vessel tone and hemodynamics [16]. Dysregulation of NO is implicated in CVD including atherogenesis and hypertension [16,67]. The T allele of the eNOS3-rs1799983 G>T SNV (Asp298Glu) was reported to reduce the enzyme activity of the eNOS [65,67,68]. Future protein–protein interaction (PPI) studies [69,70] are recommended to examine the effect of eNOS3-rs1799983 on the enzyme catalytic activity and eNOS3 PPI interactions network. Our results showed that the eNOS3-rs1799983 G>T SNV genotype distribution was significantly different in cases with normal and those with elevated lipid profile (Table 14). In addition, there was a significant difference in eNOS3-rs1799983 G>T SNV genotype distribution between obese cases and non obese cases (Table 14). Results also showed that the T allele eNOS3-rs1799983 G>T SNV is associated with CAD (Table 12). The study by Luo et al., 2019, has also reported the association of NOS3 rs1799983 with CAD [71], and that the T allele of the rs1799983 is associated with reduced NO levels and abnormal levels of lipids in blood leading to the development of CAD [71]. Population based studies in different countries have also demonstrated the association of eNOS3-rs1799983 G>T with coronary slow flow phenomenon in Iranians and CAD in Egyptians [68,72]. The eNOS has important roles in regulation of obesity and the general metabolism by stimulating mitochondrial biogenesis and activity in adipose tissues [73]. As mentioned in our results, there was a significant difference in eNOS3-rs1799983 G>T SNV between patients with and without T2D (Table 14). The normal NO signaling associates with glucose homeostasis mediated by insulin [16]. A few years ago, Chen et al., 2016, indicated that polymorphism at the eNOS3 is associated with diabetes and diabetic nephropathy in Chinese and Iranian populations [74,75]. Another study reported that overexpression of the eNOS3 does not ameliorate systemic insulin resistance [73]. The role of the eNOS3 polymorphism in the development of insulin resistance and T2D require further investigations. Results indicated that the eNOS3-rs1799983 G>T SNV genotype distribution was significantly different in cases with and those without hypertension and MI (Table 14). NO is a potent vasodilator, and it suppresses the activation and adhesion of platelets to the vascular endothelium [16]. Moreover, NO targets the mitochondria and reduces the generation of the reactive oxygen species (ROS) and decrease the mitochondrial calcium [16]. The accumulation of ROS and calcium are implicated in the progression of CAD [16,76]. Furthermore, NO protects against MI by contributing in ischemic preconditioning [16], and the physiologic levels of NO enhances lipolysis of adipocyte and oxidation of fatty acid in skeletal muscles as well as in myocardium [16,77].

## 5. Conclusions

Taken together, our results showed that the MTHFR 677C>T, KLF-14 rs972283 G>A, miRNAs27a rs895819 A>G and eNOS3-rs1799983 G>T SNVs are associated with CAD susceptibility in the Saudi population. To the best of our knowledge, this is the first study that has comprehensively evaluated the association of the eNOS, KLF-14, MTHFR, and miRNAs27a SNVs with the risk of coronary artery diseases in the given population. These findings require verification in future large-scale population-based studies before these loci are used for prediction and identification of individuals at risk to CAD. In addition, further studies merging these SNVs to evaluate their combination effects on CVD susceptibility are recommended: such studies will greatly improve the diagnosis of CAD and the prediction of individuals who are susceptible to this disease.

## Figures and Tables

**Table 1:** ARMS primers for KLF-14, miRNAs27a, *MTHFR* 677 and eNOS3 SNVs.

Direction	Sequence	Product Size	AT
ARMS primers of KLF14 rs972283 genotyping			
KLF-14 FO	5′-GTCATAGGTCAAACAGCTAGATATTGGGT-3′	437 bp	60 °C
KLF-14RO	5′-TCTACAGGACCAACTCAAATTATGAGGT-3′		
KLF-14 FI-(G allele)	5′-TCATTGTATACTTGGAAAAAATCCTACATG-3′	274 bp	
KLF14 RI-(A allele)	5′-TATGTAAAAATAAGTATGCGCCATGCCT-3′	221 bp	
ARMS primers of miRNAs27a rs895819 genotyping			
miRNAs27aFO	5′-GGCTTGACCCCTGTTCCTGCTGAACT-3′	353 bp	63 °C
miRNAs27aRO	5′-TTGCTTCCTGTCACAAATCACATTGCCA-3′		
miRNAs27a FI-(G allele)	5′-GGAACTTAGCCACTGTGAACACGACTTTGC-3′	184 bp	
miRNAs27a-(A allele)	5′-CTTAGCTGCTTGTGAGCAGGGTCCCCA-3′	226 bp	
ARMS primers for *MTHFR* 677 C>T rs1801133 genotyping			
MTHFR677FO	5′-AAGCATATCAGTCATGAGCCCAGCC-3′,	224 bp	58°C
MTHFR677RO	5′-GGGAAGAACTCAGCGAACTCAGCAC-3′		
MTHFR677 FI-C	5′-AGGAGAAGGTGTCTGCGGGCGT-3′	101 bp	
MTHFR677 RI-T	5′-AAGAAAAGCTGCGTGATGATGAAATAGG-3′	177 bp	
ARMS primers for eNOS3-rs1799983 (Glu298Asp) G>T genotyping			
eNOS3-FO	5′-AGCCTCGGTGAGATAAAGGATG-3′	701 bp	60°C
eNOS3-RO	5′-CCTGGACCTGCTCTGATTGTC-3′		
eNOS3-FI-G	5′-GCTGCTGCAGGCCCCAGATAAG-3′	475 bp	
eNOS3-RI-T	5′-GCAGAAGGAAGAGTTCTGGGAGA-3′	271 bp	

**Table 2:** Demographic features of CAD patients and controls.

Parameters	CAD Controls		Controls	
	N = 125	%	N = 125	%
Male	84	67.2	76	74.75
Female	41	32.8	49	47.75
Age < 50	95	76	70	68.75
Age > 50	30	24	55	53.75
Cholesterol ≤ 200 (mg/dL)	60	48		
Cholesterol > 200 (mg/dL)	65	52		
LDL ≤ 100 (mg/dL)	74	59.2		
LDL > 100 (mg/dL)	51	40.8		
HDL ≤ 40 (mg/dL)	55	44		
HDL > 40 (mg/dL)	70	56		
TGL ≤ 150 (mg/dL)	80	64		
TGL > 150 (mg/dL)	45	36		
Creatinine < 1.35 mg/dL	74	59.2		
Creatinine > 1.35 mg/dL	51	40.8		
C-reactive protein < 10 mg/L	53	42.4		
C-reactive protein > 10 mg/L	72	57.6		
Hypertension	52	41.6		
No hypertension	73	58.4		
T2D	55	44		
No T2D	70	56		
Smoking (Yes)	70	56		
Smoking (No)	55	44		
Obesity	40	32		
No Obesity	83	66.4		
Myocardial infarction	72	57.6		
No Myocardial infarction	53	42.4		

**Table 3:** Association between MTHFR 677C>T genotypes between CAD patients and healthy controls.

Subjects	N=	GG	GA	AA	Df	X^2^	G	A	*p* Value
Cases	116	67 (57.75%)	44 (37.93%)	14 (12.06%)	2	6.11	0.69	0.31	0.045
Controls	125	79 (63.2%)	31 (24.8%)	06 (4.48%)			0.83	0.17	

**Table 4:** Association of MTHFR 677 C>T genotypes with CAD Susceptibility.

Genotypes	Healthy Controls (N = 116)	CAD Cases (N = 125)	OR (95% CI)	Risk Ratio (RR)	*p* Value
Codominant inheritance model					
MTHFR-CC	79	67	1 (ref.)	1 (ref.)	
MTHFR-CT	31	44	1.67 (0.9529 to 2.9393)	1.30 (0.9618 to 1.7817)	0.073
MTHFR-TT	06	14	2.75 (1.0018 to 7.5558)	1.80 (0.9084 to 3.5814)	**0.049**
Dominant inheritance model					
MTHFR-CC	79	67	1 (ref.)	1 (ref.)	
MTHFR-(CT+TT)	37	58	1.84 (1.0930 to 3.1257)	1.38 (1.0367 to 1.8618)	**0.021**
Recessive inheritance model					
MTHFR-(CT+TT)	110	111	1 (ref.)	1 (ref.)	
MTHFR-TT	06	14	2.31 (0.8574 to 6.2359)	1.65 (0.8385 to 3.2829)	**0.09**
Allele					
MTHFR-C	189	178	1 (ref.)	1 (ref.)	
MTHFR-T	42	72	1.82 (1.1816 to 2.8041)	1.39 (1.0777 to 1.8130)	**0.006**

Statistically significant *p*-values with in bold.

**Table 5:** The association of MTHFR 677 C>T genotypes with CAD patients characteristics.

Parameters	N=	GG	GA	AA	X^2^	Df	*p* Value
Male	84	45	30	09	0.07	2	0.96
Female	41	22	14	05			
Age < 50	95	48	38	09	4.34	2	0.11
Age > 50	30	19	06	05			
Cholesterol ≤ 200 (mg/dL)	60	23	27	10	11.24	2	**0.003**
Cholesterol > 200 (mg/dL)	65	44	17	04			
LDL-C ≤ 100 (mg/dL)	74	30	34	10	12.60	2	**0.0018**
LDL > 100 (mg/dL)	51	37	10	04			
HDL-C ≤ 40 (mg/dL)	55	28	19	08	1.13	2	0.560
HDL-C > 40 (mg/dL)	70	39	25	06			
TGL ≤ 150 (mg/dL)	80	40	33	07	4.04	2	0.136
TGL > 150 (mg/dL)	45	27	11	07			
Creatinine < 1.35 mg/dL	74	36	30	08	2.32	2	0.313
Creatinine > 1.35 mg/dL	51	31	14	06			
C-reactive protein < 10 mg/L	53	23	20	10	6.69	2	**0.033**
C-reactive protein > 10 mg/L	72	44	24	04			
Hypertension	52	19	27	06	11.92	2	**0.002**
No hypertension	73	48	17	08			
T2D	55	36	11	08	10	2	**0.0067**
No T2D	70	31	33	06			
Smoking (Yes)	70	31	34	05	13	2	**0.005**
Smoking (No)	55	36	10	09			
Obesity	40	15	17	08	7.8	2	**0.020**
No Obesity	83	52	27	06			
Myocardial infarction (MI)	72	29	37	6	19.51	2	**0.0001**
No Myocardial infarction (MI)	53	38	7	8			

Statistically significant *p*-values in bold.

**Table 6:** Association of CAD patients and controls for KLF-14 rs972283 G>A genotypes.

Subjects	N=	GG	GA	AA	Df	X^2^	G	A	*p* Value
Cases	125	58 (46.4%)	28 (22.4%)	39 (31.2%)	2	6.43	0.57	0.43	0.045
Controls	125	50 (40%)	60 (48%)	15 (12%)			0.64	0.36	

**Table 7:** Association of KLF-14 rs972283 G>A genotypes with risk to CAD.

Genotypes	Healthy Controls (N = 125)	CAD Cases (N = 125)	OR (95% CI)	Risk Ratio(RR)	*p* Value
Codominant inheritance model					
KLF14-GG	50	58	1 (ref.)	1 (ref.)	
KLF14-GA	60	28	0.40 (0.2237 to 0.7234)	0.67 (0.5297 to 0.8704)	**0.002**
KLF14-AA	15	39	2.24 (1.1070 to 4.5383)	1.66 (1.0358 to 2.6817)	**0.024**
Dominant inheritance model					
KLF14-GG	50	58	1 (ref.)	1 (ref.)	
KLF14-(GA+AA)	75	67	0.77 (0.4663 to 1.2718)	0.87 (0.6787 to 1.1320)	**0.30**
Recessive inheritance model					
KLF14-(GG+GA)	110	86	1 (ref.)	1 (ref.)	
KLF14-AA	15	39	3.32 (1.7207 to 6.4275)	2.02 (1.2914 to 3.1608)	**0.0004**
Allele					
KLF14-G	160	144	1 (ref.)	1 (ref.)	
KLF14-A	90	106	1.30 (0.9130 to 1.8758)	1.14 (0.9520 to 1.3800)	0.1431

Statistically significant *p*-values in bold.

**Table 8:** The association of KLF-14 rs972283 G>A genotypes with CAD patient characteristics.

Parameters	N=	GG	GA	AA	X^2^	Df	*p* Value
CAD Patients	125	58	28	39			
Male	84	34	20	30	3.84	2	0.146
Female	41	24	08	09			
Age < 50	95	46	20	29	0.73	2	0.69
Age > 50	30	12	8	10			
Cholesterol ≤ 200 (mg/dL)	60	21	21	18	11.46	2	0.0032
Cholesterol > 200 (mg/dL)	65	37	07	21			
LDL ≤ 100 (mg/dL)	74	25	22	27	12.2	2	0.0022
LDL > 100 (mg/dL)	51	33	06	12			
HDL ≤ 40 (mg/dL)	55	43	18	21	2.67	2	0.263
HDL > 40 (mg/dL)	70	15	10	18			
TG ≤ 150 (mg/dL)	80	35	13	32	9.6	2	0.008
TG > 150 (mg/dL)	45	23	15	07			
Creatinine < 1.35 mg/dL	74	30	16	28	3.95	2	0.138
Creatinine > 1.35 mg/dL	51	28	12	11			
C-reactive protein < 10 mg/L	53	13	15	25	18.44	2	0.0001
C-reactive protein > 10 mg/L	72	45	13	14			
Hypertension	52	21	13	18	1.3	2	0.522
No hypertension	73	37	15	21			
T2D	55	16	17	22	11.95	2	0.0002
No T2D	70	42	11	17			
Smoking (Yes)	70	26	16	28	6.9	2	0.031
Smoking (No)	55	32	12	11			
Obesity	40	13	11	16	4.59	2	0.100
No Obesity	83	45	17	23			
Myocardial infarction (MI)	72	31	23	18	9.41	2	0.009
No Myocardial infarction (MI)	53	27	5	21			

**Table 9:** Association of *miRNAs27a* rs895819 A>G genotypes in CAD cases and controls.

Subjects	N=	AA	AG %	GG %	Df	X^2^	A	G	*p* Value
Cases	125	65 (52%)	53 (42.4%)	7 (5.6%)	2	6.83	0.73	0.27	0.032
Controls	125	56 (44.8%)	50 (40%)	19 (15.2%)			0.65	0.35	

**Table 10:** Association of *miRNAs27a* rs895819 A>G gene variations with CAD susceptibility.

Genotypes	Healthy Controls (N = 125)	CAD Cases (N = 125)	OR (95% CI)	Risk Ratio (RR)	*p*-Value
Codominant Dominant inheritance model					
*miRNA27a*-AA	56	65	1 (ref.)	1 (ref.)	
*miRNA27a*-GA	50	53	0.91 (0.5669 to 1.5038)	0.95 (0.7258 to 1.2501)	0.738
*miRNA27a*-GG	19	07	0.31 (0.1243 to 0.8104)	0.63 (0.4682 to 0.8567)	0.016
Dominant inheritance model					
*miRNA27a*-AA	56	65	1 (ref.)	1 (ref.)	
*miRNA27a*-(GA+GG)	69	60	0.74 (0.4556 to 1.2320)	0.86 (0.6735 to 1.1116)	0.25
Recessive inheritance model					
*miRNA27a*-(AA+GA)	106	118	1 (ref.)	1 (ref.)	
*miRNA27a*-GG	19	07	0.33 (0.1338 to 0.8184)	0.62 (0.4801 to 0.8076)	0.016
Allele					
*miRNA27a*-A	162	183	1 (ref.)	1 (ref.)	
*miRNA27a*-G	88	67	0.67 (0.4601 to 0.9873)	0.82 (0.6927 to 0.9876)	0.04

**Table 11:** Association of miRNA-27a rs895819 A>G genotypes with respect to the clinical feature of coronary artery disease patients.

Parameters	N=	AA	GA	GG	X^2^	Df	*p*-Value
	125	65	53	7			
Male	84	47	33	4	1.68	2	0.43
Female	41	18	20	03			
Age < 50	95	49	43	3	1.68	2	0.431
Age > 50	30	16	10	4			
Cholesterol ≤ 200 (mg/dL)	60	28	31	1	6.6	2	0.046
Cholesterol > 200 (mg/dL)	65	37	22	6			
LDL ≤ 100 (mg/dL)	74	47	23	4	10.12	2	0.006
LDL > 100 (mg/dL)	51	18	30	3			
HDL ≤ 40 (mg/dL)	55	30	23	2	0.81	2	0.66
HDL > 40 (mg/dL)	70	35	30	5			
TG ≤ 150 (mg/dL)	80	38	41	1	12.48	2	0.0019
TG > 150 (mg/dL)	45	27	12	6			
Creatinine < 1.35 mg/dL	74	44	26	4	4.21	2	0.121
Creatinine > 1.35 mg/dL	51	21	27	3			
C-reactive protein < 10 mg/L	53	29	22	2	0.7	2	0.70
C-reactive protein > 10 mg/L	72	36	31	5			
Hypertension	52	15	33	4	19.01	2	0.0001
No hypertension	73	50	20	3			
T2D	55	17	34	4	17.63	2	0.0001
No T2D	70	48	19	3			
Smoking (Yes)	70	30	35	5	5.4	2	0.06
Smoking (No)	55	35	18	2			
Obesity	40	18	20	2	1.39	2	0.499
No Obesity	85	47	33	5			
Myocardial infarction (MI)	72	46	24	2	10.32	2	0.005
No Myocardial infarction (MI)	53	19	29	5			

**Table 12:** Association of eNOS3-rs1799983 G>TSNV between cases and controls.

Subjects	N=	GG	GA	AA	Df	X^2^	G	A	*p* Value
Cases	125	38 (30.4%)	82 (65.6%)	5 (4%)	2	14.57	0.63	0.37	0.0007
Controls	125	64 (50.4%)	52 (41.6%)	09 (8%)			0.71	0.29	

**Table 13:** Association of eNOS3-rs1799983 (894G>T) gene variations with CAD susceptibility.

Genotypes	Healthy Controls (N = 125)	CAD Cases (N = 125)	OR (95% CI)	Risk Ratio(RR)	*p*-Value
Codominant inheritance model					
eNOS3-GG	64	38	1 (ref.)	1 (ref.)	
eNOS3-GT	52	82	2.65 (1.5619 to 4.5162)	1.61 (1.2468 to 2.0969)	0.0003
eNOS3-TT	09	05	0.93 (0.2920 to 2.9985)	0.97 (0.644 to 1.3800)	0.74
Dominant inheritance model					
eNOS3-GG	64	38	1 (ref.)	1 (ref.)	
eNOS3-(GT+TT)	61	87	2.40 (1.4319 to 4.5162)	1.52 (1.1931 to 1.9424)	0.0009
Recessive inheritance model					
eNOS3-(GG+GT)	116	120	1 (ref.)	1 (ref.)	
eNOS3-TT	09	05	0.53 (0.1748 to 1.6503)	0.76 (0.5067 to 1.1538)	0.277
Allele					
G	180	158	1 (ref.)	1 (ref.)	
T	70	92	1.49 (1.0268 to 2.1834)	1.23 (1.0062 to 1.5096)	0.036

**Table 14:** Association of eNOS3-rs1799983 (Glu298Asp) G>T genotypes with respect to CAD case characteristics.

Parameters	N=	GG	GA	AA	X^2^	Df	*p* Value
	125	38	82	5			
Male	84	30	51	3	3.43	2	0.18
Female	41	8	31	2			
Age < 50	95	25	66	4	3.12	2	0.210
Age > 50	30	13	16	1			
Cholesterol ≤ 200 (mg/dL)	60	25	33	2	6.92	2	0.031
Cholesterol > 200 (mg/dL)	65	13	49	3			
LDL ≤ 100 (mg/dL)	74	28	44	2	5.11	2	0.077
LDL > 100 (mg/dL)	51	10	38	3			
HDL ≤ 40 (mg/dL)	55	20	31	4	5.06	2	0.079
HDL > 40 (mg/dL)	70	18	51	1			
TG ≤ 150 (mg/dL)	80	30	49	01	8.43	2	0.014
TG > 150 (mg/dL)	45	8	33	4			
Creatinine < 1.35 mg/dL	74	25	47	2	1.57	2	0.45
Creatinine > 1.35 mg/dL	51	13	35	3			
C-reactive protein < 10 mg/L	53	24	25	04	14.36	2	0.0008
C-reactive protein > 10 mg/L	72	14	57	01			
Hypertension	52	26	23	3	18.13	2	0.0001
No hypertension	73	12	59	2			
T2D	55	10	42	03	7.08	2	0.029
No T2D	70	28	40	02			
Smoking (Yes)	70	22	45	03	0.13	02	0.930
Smoking (No)	55	16	37	02			
Obesity	40	20	19	01	10.07	2	0.0047
No Obesity	85	18	63	04			
Myocardial infarction (MI)	72	30	38	4	12.37	2	0.0021
No Myocardial infarction (MI)	53	08	44	01			

## Data Availability

All the data associated with the current study has been presented in this manuscript.
